# Influenza Treated with Pyrenol

**Published:** 1907-07

**Authors:** 


### Influenza Treated with Pyrenol.

Dr. M. Steiner writes from the Ducal Hospital and Sanitarium at Altenburg (Director, Privy Councillor Neutzenadel) that a remedy for influenza must meet three requirements: (1) it must effect subjective improvement, i. e. depress temperature and relieve pains; (2) it must be innocuous; and (3) it must as far as possible prevent complications. He applies these tests to pyrenol (benzoyl-thymyl-sodium-benzoyl-oxy-ben-zoate), which has lately been recommended for influenza in about thirty publications.

That pyrenol is antipyretic and analgesic must be conceded. Thus, reports from the Augusta Hospital in Berlin and the General Hospital in Vienna show that its antithermic effect is slow and steady, accompanied by subjective betterment; there is no exhausting diaphoresis under its use, as there is with antipyretics which act suddenly and rapidly. As to innocuousness, hardly a single writer fails to record the improvement in the appetite and the general absence of gastrointestinal disturbances under its use. The sphygmometric experiments of Cowl showed total absence of any untoward action; to the contrary, the drug is a cardiotonic,—a most valuable property in influenza. Nor does pyrenol ever irritate the kidneys.

Steiner's own observations with pyrenol began in 1903, were especially numerous in the epidemic of 1904-5, and were confirmed in the autumns of 1905 and 1906. He has records of 86 cases treated with pyrenol and 20 control cases which got other treatment. Cases in the prodromal stage of influenza took 15 grains pyrenol thrice daily or 7½ grains six times daily; when the patient could not swallow tablets, 5% solution was given. If symptoms were already well developed and severe, the dose was 15 grains four times daily or half the dose every three hours, given until the first signs of improvement occurred and then reduced to 45 grains daily, and continued till he got out of bed. Cardiac lesions, nephritis or liver trouble were no contra-indications.

The third test of pyrenol is the absence of complications. Of the patients seen in the prodromal stage the great majority remained under treatment only for from a few days to a week; and not one developed pneumonia. In almost all pyrenol was the only treatment required. In the first two days large hot compresses were applied for two hours twice daily to the trunk to promote diaphoresis, and pyrenol seemed to do especially well under this treatment. Vigorous rubbing of the body after the sweating seemed to put the patient in good condition for some hours.

Of the patients seen only when the malady was already advanced, pneumonia was positively diagnosticated in eight. The dose of pyrenol was usually increased by 15 grains daily, to control fever and pain more rapidly. There was not a single death. As temperature and pulse were kept in moderate bounds, the subjective symptoms were bearable and the course of the disease was very favorably modified. Cough during the febrile stage was treated exclusively with pyrenol.

Pyrenol was very helpful in the influenza of children. In young subjects almost all antipyretics, even in small doses, are apt to depress the temperature to subnormal, with collapse lasting perhaps for hours; so we are glad to have a remedy which slowly and gradually lessens the fever and does not depress the heart. Children from six months to eight years got, in accordance with age, one or two teaspoonfuls of a 2 to 4% pyrenol solution in raspberry syrup, three or four times a day. Its antifebrile action lasted longer in children than in adults, so less frequent administrations were necessary.

To conclude: pyrenol in influenza depresses the fever gently and gradually, with very light sweating and an increase in the general well-being. It relieves the pains in the joints, muscles, tendons and fasciæ, as well as those of the pleural and intercostal nerves. It is a powerful expectorant, loosening the mucous and diminishing secretion.

In the *Berliner Klin,* Wochenschr., Feb. 11, 1907, Dr. Braeunig refers to Steiner's paper and adds that it tells nothing new, for the beneficial effect of pyrenol in influenza and pertussis is well known. Braeunig also found it to do good service in these cases, as well as in the hectic fever of phthisis in which—given at the right time—it forestalls the high evening temperatures without causing the annoying diaphoresis which follows other antipyretics.

